# Effect of platelet-derived growth factor (PDGF-BB) and bone morphogenic protein 2 (BMP-2) transfection of rBMSCs compounded with platelet-rich plasma on adipogenic differentiation

**DOI:** 10.1590/1414-431X20209944

**Published:** 2020-12-09

**Authors:** Jin Sun, Xin Jiang, Junnan Luo, Liheng Zhao, Zuhua Xu, Wende Xiao

**Affiliations:** 1 Department of Orthopedics, Huizhou Third People's Hospital, Guangzhou Medical University, Huizhou, Guangdong, China; 2 Department of Orthopedics, Guangzhou First People's Hospital, School of Medicine, South China University of Technology, Guangzhou, Guangdong, China

**Keywords:** Platelet-derived growth factor, Bone morphogenic protein, Platelet-rich plasma, Adipogenic

## Abstract

The aim of this study was to inhibit adipogenic differentiation by transfecting two growth factors, platelet-derived growth factor (PDGF-BB) and bone morphogenic protein 2 (BMP-2), into modified rat bone marrow mesenchymal stem cells (rBMSCs) and then compounded with platelet-rich plasma (PRP). To achieve rBMSCs, the osteoporosis model of rats was established, and then the rBMSCs from the rats were isolated and identified. Co-transfection of rBMSCs with PDGF-BB-GFP and BMP-2 and detection of PDGF-BB/BMP-2 expression in transfected BMSCs was assessed by qRT-PCR and western blot, respectively. Moreover, the effect of the two growth factors transfection of rBMSCs on adipogenic differentiation was evaluated by oil red O staining and western blot, respectively. Finally, construction of the two growth factors transfection of rBMSCs compounded with PRP and detection of adipogenic differentiation were assessed by oil red O staining, CCK-8, and western blot, respectively. *In vitro* studies revealed that the two growth factors transfection of rBMSCs compounded with PRP promoted cell viability and inhibited adipogenic differentiation and could be promising for inhibiting adipogenic differentiation.

## Introduction

At present, China's population over the age of 60 has exceeded 210 million (approximately 15.5% of the population), and the population over 65 is nearly 140 million (approximately 10.1% of the total population) (1,2). Fractures caused by osteoporosis are one of the leading causes of disability and death in elderly patients. According to incomplete statistics, the total number of osteoporosis patients in China has reached 7% of the total population, and the incidence of fractures due to osteoporosis has exceeded 9% each year (3,4).

It is well known that bone marrow mesenchymal stem cells (BMSCs) can not only differentiate into osteoblasts, but also into adipocytes. In recent years, it has been found that the balance between osteogenic differentiation and adipogenic differentiation of BMSCs maintains a steady state of bone metabolism and fat metabolism in the bone marrow (5). Fat in the bone marrow is not only a tissue for passive energy storage, but also an endocrine organ that regulates energy metabolism, inflammation, and bone formation and development. Adipocytes can secrete many hormones and cytokines. These molecules are commonly called adipocytokines, including leptin, tumor necrosis factor-α (TNF-α), plasminogen activator, inhibitor-1 (plasminogen activator inhibitor-1 (PAI-1)), interleukin-6, resistin, adiponectin, etc. (6).

Previous studies have found that bone morphogenic protein (BMP-2) can promote the expansion and osteogenic differentiation of BMSCs from osteoporotic bone marrow, as well as repair osteoporotic bone defects (7,8). However, inadequate vascularization of tissue engineering materials after implantation in the body has become one of the major bottlenecks hindering its clinical application. Platelet-derived growth factor (PDGF-BB) plays an important role in regulating bone tissue reconstruction, promotes local blood vessel formation, and plays an important regulatory role in fracture healing (9). In addition, platelet-rich plasma (PRP) can promote the repair and regeneration of osteoporotic fractures and bone defects, promote osteoporotic bone regeneration, inhibit adipogenesis, as well as inhibit osteoclast formation (10,11).

Difficulty in healing osteoporotic fractures is closely related to the enhancement of the adipogenic capacity of BMSCs. Therefore, inhibiting the adipogenic capacity of BMSCs is very important for promoting the healing of osteoporotic fractures. In the present study, we explored the inhibition of adipogenic differentiation by transfecting two growth factors, PDGF-BB and BMP-2, into modified rBMSCs and then compounded with PRP.

## Material and Methods

### Animals

All animal experiments were approved by Guangdong Medical Experimental Animal Center. All animals were maintained at the animal facility of Guangdong Medical Experimental Animal Center under protocols approved by the Institutional Animal Care and Use Committee (IACUC).

### Establishment of rat osteoporosis model

Eight adult female Sprague-Dawley rats (8-week-old, 480-550 g) were randomly separated into Sham group (n=4) and osteoporosis model (OP) group (n=4). After abdominal anesthesia with 3% pentobarbital sodium (40 mg/kg), the rats were positioned on their backs and the fur was removed from the abdomen. Under sterile conditions, the skin and muscular layer were incised along the midline of the groin to expose the abdominal cavity. In the OP group, the uterus was separated and both ovaries were removed. In the Sham group, only about 1 g of adipose tissue around the ovaries was removed, and the ovaries were retained. The rest of the operation was the same for both groups. The wound was cleaned, the skin and muscle layers sutured, and after the rat awoke, it was returned into the clean cage in the rearing room for 3 months. The state and death of the rats was examined daily and their body weight was measured weekly.

### Micro-computed tomography (micro-CT)

Rats were euthanized 12 weeks after surgery and used for micro-CT evaluation. Animal micro-CT scanners (eXplore Locus, GE Healthcare Biosciences, UK) were used to assess the reconstructed form of the cortex. The sample was scanned with a 55 kVp energy setting, an intensity of 145 mA, a 200 ms acquisition time, and a frameless average in high-resolution mode that provided a resolution of 12 µm. After the micro-CT scan, the bone mineral densities (BMDs) of the defect area were identified.

### rBMSCs morphology

The two groups of euthanized rats were immersed in 75% ethanol for 5 min. The hind limbs of both sides of the rats were separated under sterile conditions, and the skin, fascia, and muscle were removed and placed in a petri dish filled with PBS (Gibco Co., USA). The femoral and tibia ends of the epiphysis were cut off, and the bone marrow cavity was repeatedly washed with DMEM low-sugar medium containing 10% FBS (Gibco Co.) with a 10 mL syringe until the bone became white. The cell suspension was collected and filtered through a 200-µm-mesh sieve to prepare a single-cell suspension. Then, the suspension was centrifuged at 1000 *g* at 25°C for 5 min, the supernatant was discarded, the cell pellet resuspended by adding 10% FBS DMEM (Gibco Co.) low-sugar medium, and the cell density adjusted to about 5×10^6^ cells/mL. The inoculate was placed in a T25 culture flask at 37°C with 5% CO_2_ in an incubator. The liquid was changed every 2∼3 d. When the plate was covered with a monolayer of cells, cells were collected after trypsinization and passaged at a ratio of 1:2. The morphological changes of rBMSCs cells in Sham and OP groups were observed with an inverted phase contrast microscope (Olympus, Japan).

### Identification of rBMSCs surface markers by flow cytometry

Trypsinase was added to the P3 generation of rBMSCs and 5 suspensions of 200 µL were prepared with a cell density of 2×10^6^ cells/mL. CD29, CD34, or CD90 goat anti-rat monoclonal antibodies (5 µL each) were added to the suspensions. CD29, CD34, or CD90 goat anti-rat monoclonal antibodies were used as a positive control, and an equal amount of PBS was used as a negative control. A secondary antibody was added, and the cell was left at 4°C for 30 min. The cells were resuspended, and the cell surface markers were analyzed by flow cytometry detection (Accuri C6, Becton, Dickinson and Co., USA).

### rBMSCs infected with recombinant adenovirus

The cells were collected in the logarithmic growth phase, counted, resuspended with complete medium. The cell concentration was adjusted to 1×10^5^ cells/mL, 6-well plates were inoculated, 2-mL cell suspension was added to each well, and then incubated at 37°C, 5% CO_2_ overnight. The original medium in the culture well was aspirated, and 1 mL DMEM low-sugar medium and polybrene were added to a final concentration of 5 μg/mL. A corresponding volume of virus stock solution (Ad-PDGF-BB-GFP and Ad-BMP2-GFP, MesGen Biotech, China) at MOI=50 was added, shaken well, and incubated at 37°C, 5% CO_2_ for 4 h. Then, 1 mL of DMEM low-sugar medium was added to the culture wells and the wells were incubated overnight at 37°C and 5% CO_2_. The day after infection, the virus-containing culture medium was aspirated, 2 mL of fresh medium was added, and the culture was incubated for 48 h at 37°C and 5% CO_2_.

### qRT-qPCR

Total RNA was extracted by nano-magnetic beads using a MagBeads Total RNA Extraction Kit (TIANGEN, China). Target gene specific primers used are shown in Table 1. TB green qPCR Master Mix (Takara Bio Inc., Japan) was used for fluorescence quantitative detection according to the manufacturer's instructions. The fluorescence values were calculated by 2^-ΔΔCt^ and quantified (12,13).

**Table 1:** Primer sequences used for qRT-PCR.

Gene	Primers	5′-3′
*PPARγ*	Forward	ACAGCTGAGAGGGAAATCGTGCG
	Reverse	ACTTGCGCTCAGGAGGAGCAATG
*PDGF-BB*	Forward	CTCCATCCGCTCCTTTGA
	Reverse	TGCACTCGGTTACAG
*BMP2*	Forward	CCCCACGGAGGAGTTTATCA
	Reverse	CCTGTGTCTGTTCCCGAAAGA
*GADPH*	Forward	GGCCAGGTCATCACTATTG
	Reverse	GAGGTCTTTACGGATGTCAAC

### Western blotting

First, rBMSCs were homogenized on ice for 30 min with a RIPA buffer (Sigma-Aldrich, USA) and protease inhibitor. Next, the cell homogenate was collected by centrifugation at 12,000 *g* at 25°C for 50 min and the supernatant was stored at -20°C. A 12% SDS-polyacrylamide gel (PAGE, Sigma-Aldrich) with a Tris-glycine running buffer was used for total protein electrophoresis, and the proteins were electrically transferred onto PVDF membranes. Nonspecific binding was blocked in Tris-buffered saline with 3% bovine-serum albumin containing 0.1% Tween-20 under gentle shaking for 1 h at 25°C. Then, it was continuously cultured at 4°C overnight with the following primary antibodies: anti-proliferator-activated receptor γ (*PPARγ*), anti-bone morphogenetic protein-2 (BMP-2), and anti-platelet-derived growth factor (PDGF)-BB (all with 1:1000 dilution, Santa Cruz, USA). β-actin (1:5000, Sigma, USA) served as a loading control. The samples were incubated with horseradish peroxidase (HRP)-labeled secondary antibody (1:1000, Santa Cruz, USA) for 1 h at 25°C. The band density was quantified using the LICOR Odyssey infrared imaging system (Bio-science, USA).

### Oil red O staining

Collected logarithmic rBMSCs were resuspended in complete medium, cell concentration was adjusted to 1×10^5^ cells/mL, and 2 ml cell suspension was inoculated into 6-well plates, which were incubated overnight at 37°C with 5% CO_2_. After the cells were completely adhered, the old medium was discarded and washed three times with serum-free DMEM medium. After 14 days of adipogenic induction and differentiation, oil red O (Sigma) was used to stain the neutral lipids of rBMSCs. The rBMSCs were fixed in 4% paraformaldehyde overnight, washed with PBS, transferred to 60% isopropanol for 30 min, and then stained with freshly prepared 0.3% oil red O working solution for 3 h.

### CCK-8 assay

rBMSCs were incubated for 24 h, the original culture solution was removed, and 10% PBS was used to rinse 3 times after adding 100 μL of medium. The cells were treated different concentrations (200, 1000, 1800, and 3000×10^9^/L) of PRP compounded with PDGF-BB and BMP-2 gene-modified rBMSCs. CCK-8 solution (10 μL; Crystal Ann Biotech, China) was added to each well. The plates were cultured for 24, 48, 72, and 96 h. The corresponding absorbance was determined at a wavelength of 490 nm using an enzyme-linked immunoassay instrument (Bio-Rad Laboratories, USA).

### Statistical analysis

The software GraphPad 8.0 (GraphPad Software, USA) was used for the analysis of the data. All the experiments were performed 3 times in triplicates and results are reported as means±SD. Two-way ANOVA was used to analyze the differences between experimental groups. P<0.05 was considered significant.

## Results

### BMD in rats

**Figure 1:**
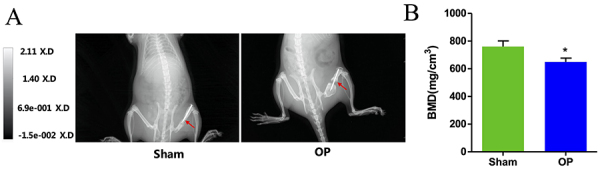
*In vivo* bone mineral density test in rats. **A**, Micro-CT cross-section analysis in the Sham and osteoporosis (OP) groups. The red arrow indicates the area of osteoporosis. **B**, Morphometric evaluation of the local bone mineral densities (BMDs) in the Sham and OP groups. Data are reported as means±SD. *P<0.01 (*t*-test).

The micro-CT cross-section of the defect area was less pronounced in the OP group than in the Sham group, as shown in Figure 1A. Significantly lower BMD was found in OP than in Sham rats (P<0.05, Figure 1B). These results indicated the successful construction of rat osteoporosis model.

### rBMSCs isolation and identification

**Figure 2:**
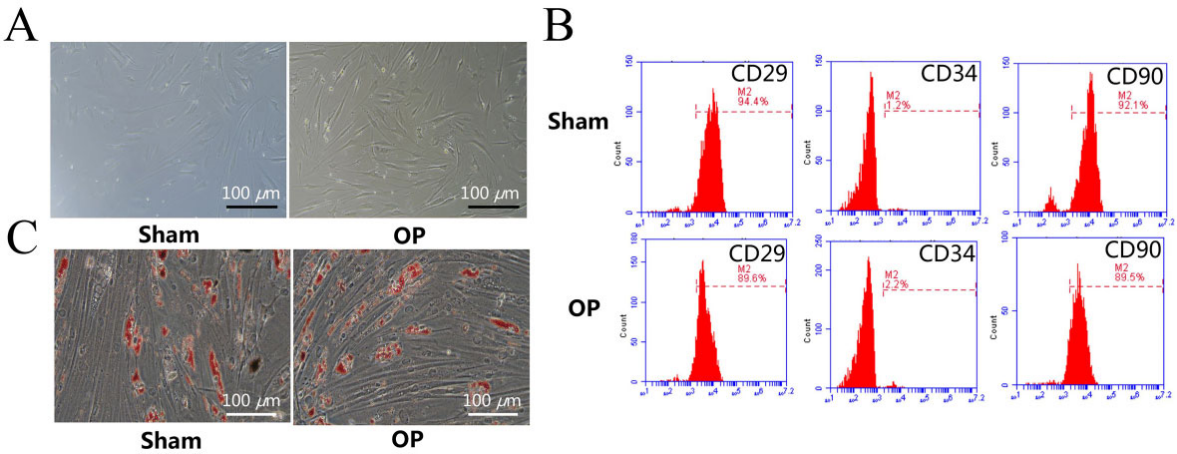
Isolation and identification of rat bone marrow mesenchymal stem cells (rBMSCs). **A**, Morphological observation of rBMSCs in Sham and osteoporosis (OP) groups (scale bar: 100 μm). **B**, Surface marker identification of rBMSCs by flow cytometry in Sham and OP groups. **C**, Oil red O staining for rBMSCs in Sham and OP groups (scale bar: 100 μm).

rBMSCs in the Sham group were spherical or elliptical and highly refractive; the cells in the OP group were completely adherent and had a spindle shape (Figure 2A). Flow cytometry showed that CD29 and CD90 were highly expressed in the OP group (89.6 and 89.5%), while CD34 expression was 2.2% (Figure 2B). These findings indicated that the obtained cells had characteristics of rBMSCs and high purity. Twenty-one days after adipogenic induction, oil red O staining microscopy showed a decreased number of fat droplets in the OP group compared to the Sham group (Figure 2C).

### rBMSCs infection by recombinant adenovirus BMP-2 and PDGF-BB

**Figure 3:**
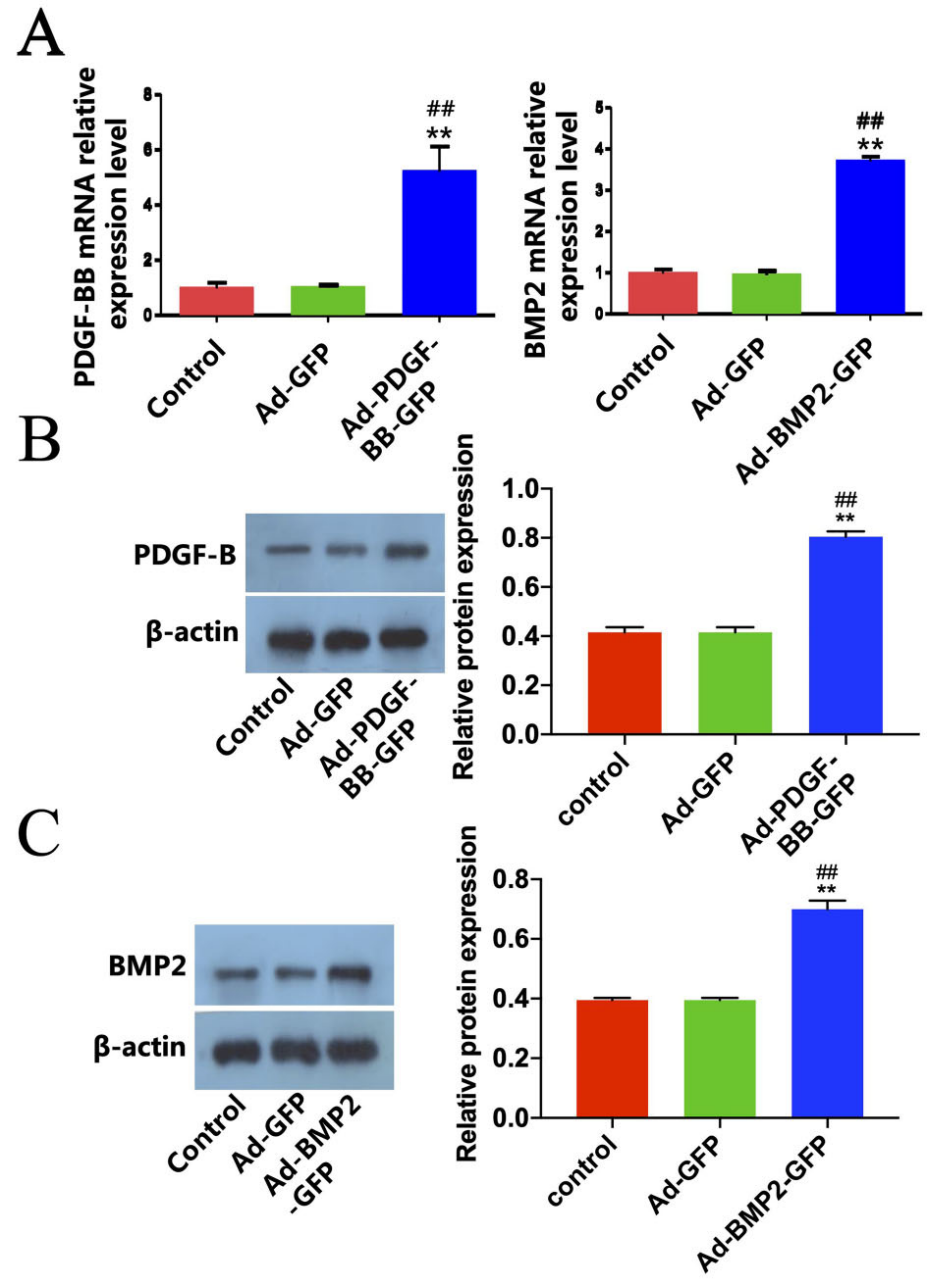
Rat bone marrow mesenchymal stem cells (rBMSCs) infection by recombinant adenovirus bone morphogenic protein 2 (BMP-2) and platelet-derived growth factor (PDGF)-BB. **A**, qRT-PCR detection of PDGF-BB and BMP-2 genes mRNA expression. **B** and **C**, Western blot detection of PDGF-BB and BMP2 protein expression after transfection. Data are reported as means±SD. **P<0.01 *vs* Control group, ^##^P<0.01 *vs* Ad-GFP group (ANOVA).

In addition, we explored the expression of BMP-2 and PDGF-BB in rBMSCs mediated by recombinant adenovirus BMP-2 and PDGF-BB. Varying Ad-BMP-2 and Ad5-PDGF-BB (5,000 virus particles/cell; MOI=50) or a control virus Ad-GFP (5,000 particles/cell; MOI=50) were added to rBMSCs and cultured for two days. Infection with both Ad-BMP-2 and Ad5-PDGF-BB virus resulted in production of mature BMP-2 and PDGF-BB mRNA and protein expressions (Figure 3A, B, and C). In addition, the amount of endogenous BMP-2 and PDGF-BB mRNA and protein expressions in the rBMSCs were decreased when mock-infected with the Ad-GFP control virus. These results demonstrated that the BMP-2 and PDGF-BB expressed after viral infection could be both processed and secreted.

### Effect of PDGF-BB and BMP-2 transfection of rBMSCs on adipogenic differentiation

**Figure 4:**
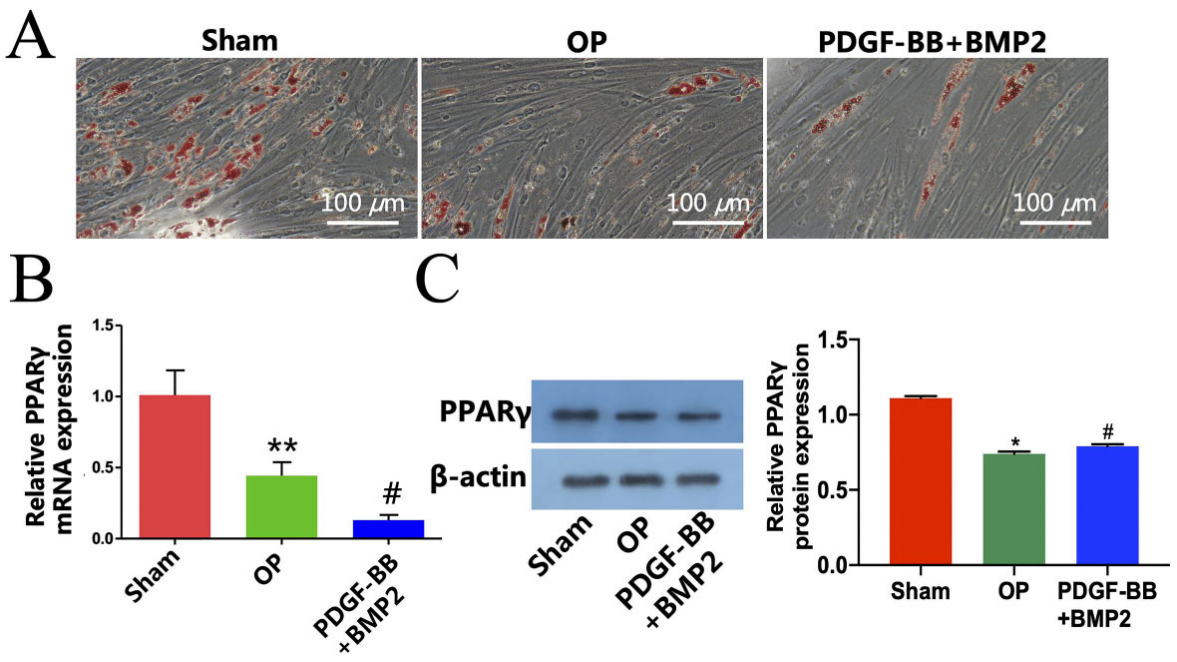
Effect of platelet-derived growth factor (PDGF)-BB and bone morphogenic protein 2 (BMP-2) transfection of rBMSCs on adipogenic differentiation. **A**, Oil red O staining for rat bone marrow mesenchymal stem cells (rBMSCs) in Sham, osteoporosis (OP), and PDGF-BB+BMP-2 groups (scale bar: 100 μm). **B**, qRT-PCR detection of adipogenic-related gene *PPARγ* in Sham, OP, and PDGF-BB+BMP2 groups. **C**, Western blot detection of adipogenic-related protein PPARγ in Sham, OP, and PDGF-BB+BMP-2 groups. Data are reported as means±SD. *P<0.05, **P<0.001 *vs* Sham group, ^#^P<0.05 *vs* OP group (ANOVA).

A decreased number of fat droplets was formed in the OP group compared to the Sham group, and PDGF-BB and BMP-2 recombinant adenovirus maintained the decrease (Figure 4A). The qRT-PCR results showed that compared with the Sham group, the mRNA expression level of *PPAR-γ* gene, an important transcription factor for adipocyte differentiation, was significantly reduced in the OP group. In addition, the decreased mRNA expression of *PPARγ* was maintained in the PDGF-BB + BMP-2 group (Figure 4B). Western blot results also showed that compared with the OP group, PDGF-BB and BMP-2 recombinant adenovirus treatment significantly inhibited the protein expression of PPARγ (Figure 4C).

### Effects of different concentrations of PRP on proliferation and adipogenic differentiation of PDGF-BB and BMP-2 transfection of rBMSCs

**Figure 5:**
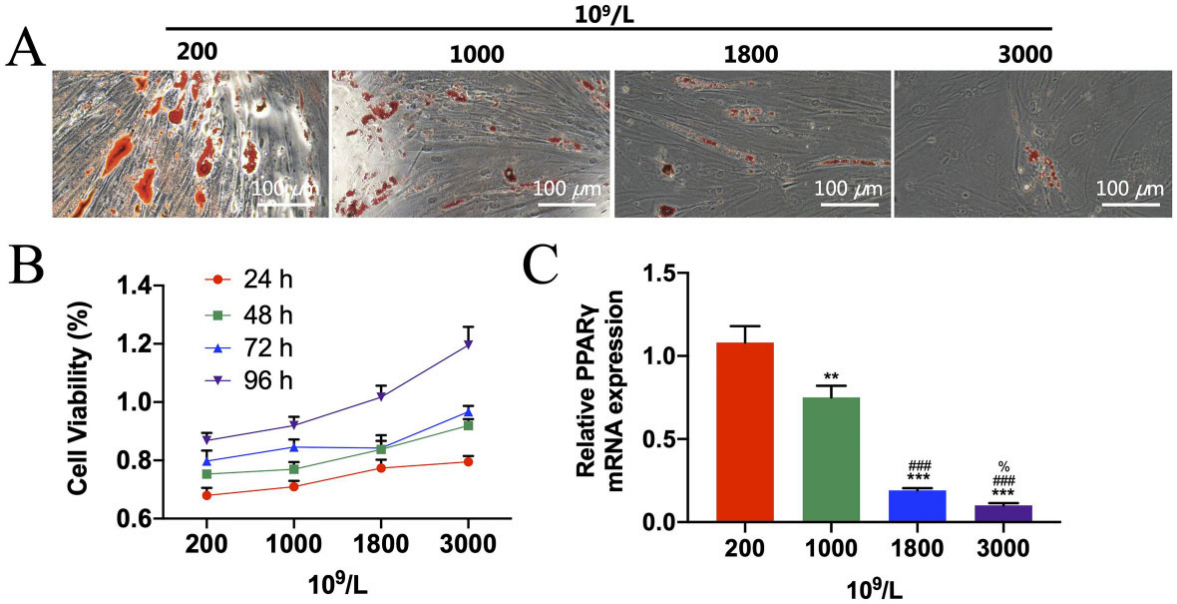
Platelet-rich plasma (PRP) promoted the cell viability and adipogenic differentiation of platelet-derived growth factor (PDGF-BB) and bone morphogenic protein 2 (BMP-2) gene-modified rat bone marrow mesenchymal stem cells (rBMSCs). **A**, Oil red O staining to detect the effect of different concentrations (200, 1000, 1800, and 3000×10^9^/L) of PRP on adipogenic differentiation of rBMSCs (scale bar: 100 μm). **B**, CCK8 detected the effects of different concentrations of PRP on rBMSCs proliferation. **C**, Effects of different concentrations of PRP on adipogenic-related gene *PPARγ* detected by qRT-PCR. Data are reported as means±SD. **P<0.01, ***P<0.001 *vs* 200×10^9^/L; ^###^P<0.001 *vs* 1000×10^9^/L; ^%^P<0.05 *vs* 1800×10^9^/L (ANOVA).

We detected the effect of different concentrations of PRP compounded with PDGF-BB and BMP-2 gene-modified rBMSCs on cell viability and adipogenic differentiation. The number of fat droplets decreased with increasing concentrations (200, 1000, 1800, and 3000×10^9^/L) of PRP (Figure 5A). The contribution of PRP to vascularization was assessed using a CCK-8 assay to examine cell viability of rBMSCs (Figure 5B). With increasing concentrations, the cell viability of rBMSCs continued to increase in a dose-dependent manner. In addition, adipocyte-related genes in rBMSCs were detected by qRT-PCR (Figure 5C). The results showed that *PPARγ* mRNA expressions were significantly decreased with increasing concentrations of PRP in a dose-dependent manner.

## Discussion

Recently, rBMSCs have become the experimental cells for much research because of they are easy to obtain and culture. There are five methods for the isolation and purification of rBMSCs: whole bone marrow natural adherent culture method, density gradient centrifugation culture method, flow cytometry technology separation culture method, immunomagnetic bead sorting technology culture method, and special microwell culture plate screening method (14–16). In the current study, a rat osteoporosis model was successfully established, rBMSCs were isolated from the rat osteoporosis model, and identified by microscopy and flow cytometry. Microscopic observation showed that a large number of osteogenic nodules were formed. The cells were highly positive for CD29 and CD90 and negative for CD34. The above characteristics are consistent with the characteristics of rBMSCs.

BMP-2 is known to be one of the most potent osteoinductive substances that enhance bone formation *in vivo* (17). Previous studies have demonstrated that BMP-2 can induce osteogenic differentiation of mesenchymal cells and new orthotopic bone formation (18–21). PDGF-BB is the most biologically effective PDGF subtype with the largest binding affinity for osteoblasts (22,23). PDGF-BB shows potential effects on cells that affect bone regeneration, such as chemotactic and mitogenic effects on osteoblasts and stimulation of type I collagen synthesis in osteoblasts, the latter being an important extracellular component of bone (24,25). PDGF-BB is an important growth factor in embryo skeletal development and is injected into the medullary cavity to accelerate fracture healing and treat osteoporosis (26,27). Several recent reports in the literature document the production of bone, *in vivo*, in response to implantation of Ad5BMP-2 transduced W20-17 mouse bone marrow stromal cells (28,29). In the present study, we explored the BMP-2 and PDGF-BB expressions in rBMSCs mediated by recombinant adenovirus BMP-2 and PDGF-BB. These results demonstrated that the BMP-2 and PDGF-BB expressed after viral infection can be both processed and secreted. Moreover, 21 days after adipogenic induction, oil red O staining microscopy showed that PDGF-BB and BMP-2 recombinant adenovirus decreased the number of fat droplets formed. The mRNA expression level of *PPAR-γ* gene was significantly reduced in the PDGF-BB + BMP-2 group. PDGF-BB and BMP-2 recombinant adenovirus treatment significantly inhibited the protein expression of *PPARγ*.

PRP contains a variety of bone growth factors, such as PDGF, TGF-β 1, TGF-β 2, IGF, EGF, FGF, and VEGF, etc. (30), that play an important role in bone regeneration, bone resorption and bone shaping (31,32). PRP can significantly promote the proliferation and osteogenic differentiation of BMSCs and adipose stem cells, and can further promote osteoblast proliferation, matrix synthesis, and angiogenesis (33,34). Choi et al. (35) studied the effect of different concentrations (1, 5, 30, 50, and 100%) of PRP on alveolar osteoblast osteogenesis *in vitro*, and found that 5% of PRP has the best osteogenesis, and 100% of PRP is non-osteogenic and toxic to cells. Liu et al. (36) studied the effects of different concentrations (2.5, 5.0, 7.5, 10.0, 12.5, and 15.0%) of PRP on the proliferation and osteogenic differentiation of cultured adipose stem cells. The results showed that 10.0 and 12.5% PRP have the best effect on osteogenic differentiation and are the most ideal application concentrations. In our present study, we found that a decreased number of fat droplets formed with increasing concentrations of PRP.

In summary, we demonstrated that the transfection of rBMSCs with two growth factors (PDGF-BB and BMP-2) and compounded with PRP could be a promising material for inhibiting adipogenic differentiation.
